# Whole-Genome Sequence of Listeria monocytogenes Serovar 1/2a Strain IZSAM_Lm_15_17439_A144, Representative of a Human Outbreak in 2008

**DOI:** 10.1128/MRA.00074-19

**Published:** 2019-09-05

**Authors:** Massimiliano Orsini, Alessandra Ordinelli, Alessandra Cornacchia, Vicdalia Acciari, Patrizia Centorame, Marina Torresi, Antonella Pompei, Maurilia Marcacci, Massimo Ancora, Marco Di Domenico, Samuel Perticara, Cesare Cammà, Adriano Di Pasquale, Antonietta Gattuso, Monica Gianfranceschi, Francesco Pomilio

**Affiliations:** a National Reference Center for Whole Genome Sequencing of Microbial Pathogens: Database and Bioinformatics Analysis, Istituto Zooprofilattico Sperimentale dell’Abruzzo e del Molise G. Caporale, Teramo, Italy; b Department of Institutional Communication, Istituto Zooprofilattico Sperimentale dell’Abruzzo e del Molise G. Caporale, Teramo, Italy; c National Reference Laboratory for *Listeria monocytogenes*, Istituto Zooprofilattico Sperimentale dell’Abruzzo e del Molise G. Caporale, Teramo, Italy; d Laboratory of Molecular Biology and Omics Science, Istituto Zooprofilattico Sperimentale dell’Abruzzo e del Molise G. Caporale, Teramo, Italy; e Istituto Superiore di Sanità, Rome, Italy; University of Delaware

## Abstract

Here, we report the genome sequence of Listeria monocytogenes serovar 1/2a strain IZSAM_Lm_15_17439_A144, isolated in Italy from a patient during a Listeria monocytogenes outbreak in 2008. This strain showed 98.9% sequence identity to a strain isolated in Canada in the same year.

## ANNOUNCEMENT

During a revision of the Listeria monocytogenes sequence database hosted by the Listeria monocytogenes Italian National Reference Center, we observed sequence similarity of a particular strain with data already published in GenBank (1). A strain sequenced in 2015 and isolated from a human case of listeriosis in 2008 showed 98.9% sequence identity with two strains (GenBank accession numbers NC_013766 and NC_013768) isolated in Canada during a large outbreak in the same year (2).

This strain was isolated during a listeriosis outbreak that occurred in 2008, from a male patient (77 years old at the time of infection) living in northern Italy who developed septicemia. The strain was isolated from peripheral blood according to the ISO 11290 procedure (3) and then frozen. It was sequenced and assembled in 2015 during the building of a sequence database aiming to collect as many Listeria monocytogenes sequences as possible from human cases. The strain was plated onto blood agar and incubated at 37°C for 24 h before nucleic acid extraction that was performed with a Maxwell 16 tissue DNA purification kit (Promega Italia Srl, Milan, Italy), according to the manufacturer’s protocol. One nanogram of genomic DNA was used for library preparation by using the Nextera XT library prep kit (Illumina, Inc., San Diego, CA), while sequencing was performed on the NextSeq 500 platform (Illumina, Inc.) using the NextSeq 500/550 mid output reagent cartridge v2, 300 cycles, and standard 150-bp paired-end reads.

A total of 2,034,523 read pairs were obtained, corresponding to a theoretical coverage of about 210×, calculated by dividing the amount of sequenced bases by the expected genome size. Quality control was performed with FastQC (http://www.bioinformatics.babraham.ac.uk/projects/fastqc/), and trimming was carried out by the command line version of the trimming tool (FASTQ positional and quality trimming) available on the Orione Web server (4) with the following parameters: 5′ trim, 8 nucleotides; 3′ minimum quality, 22; residual average quality, 24; and residual final length, 50 nucleotides. Scaffolds were assembled by using SPAdes 3.5 (5) with default parameters for Illumina 2 × 150 chemistry. Recently (2018), by comparing that preliminary assembly against GenBank, a sequence identity emerged that was higher than 98% with the abovementioned Canadian strains isolated in the same year. This finding led us to perform a sequence refinement. Scaffolds were ordered using the genome of *L. monocytogenes* 08-5923 (GenBank accession number NC_013768) as a reference using ABACAS 1.3 (6), with default settings, indicating *nucmer* as the aligner parameter. Gaps in the pseudomolecule were filled by GapFiller 1.10 (7) and FGAP 1.7 (8), both with default configurations. Finally, the draft genome was refined by Pilon (9), with default parameters, using the alignment obtained by realigning the trimmed reads to the pseudomolecule using Bowtie 2 (10), with default parameters. Searching unplaced scaffolds against the nucleotide database (1) returned some matches with the plasmid pLMR479a (GenBank accession number HG813248), which was used as a reference to order matching scaffolds. As for the chromosome, gaps were filled by GapFiller 1.10 and FGAP 1.7, and then the sequence was refined by Pilon. The presence of the origin of replication was confirmed by comparing the assembled sequence against the above-mentioned pLMR479a plasmid. For both chromosome and plasmid sequences, the circularity was confirmed by mapping (using the Bowtie 2 aligner) the paired reads against the sequences themselves and then identifying read pairs spanning the start/end of the sequence and trimming the edges of the sequences accordingly.

The whole genome that was assembled as a single circular molecule (length, 2,905,533 nucleotides; G+C content, 37.96%) was submitted to GenBank and annotated by the Prokaryotic Genome Annotation Pipeline (PGAP) pipeline (https://www.ncbi.nlm.nih.gov/genomes/static/Pipeline.html). Annotation returned 2,907 genes, 2,818 protein-coding sequences, 5 full rRNA operons, 11 pseudogenes, 5 noncoding RNAs (ncRNAs), 58 tRNAs, and 1 CRISPR array. The sequenced genome showed an overall identity against the Listeria monocytogenes 08-5923 strain genome (GenBank accession number NC_013768) of 99.85% calculated by the OrthoANI tool (11). One full-length plasmid (length, 86,632 nucleotides; G+C content, 36.98%) was detected; it showed a global identity of 100%, out of a 20-nucleotide gap near the origin of replication, toward the plasmid pLMR479a (GenBank accession number HG813248), which was detected in the above-mentioned Listeria monocytogenes 08-5923 strain (3). Although sequenced years ago, the genome sequence availability can help in clarifying mechanisms of strain circulation.

### Data availability.

Both the complete genome and plasmid sequences of this isolate have been deposited in GenBank with the accession numbers CP013919 and KU513859, respectively. Raw reads were uploaded to the SRA database under the accession number SRR8442684.

## References

[1] Benson DA, Cavanaugh M, Clark K, Karsch-Mizrachi I, Lipman DJ, Ostell J, Sayers EW 2013 GenBank. Nucleic Acids Res 41:D36–D42. doi:10.1093/nar/gks1195. 23193287 PMC3531190

[2] Gilmour MW, Graham M, Van Domselaar G, Tyler S, Kent H, Trout-Yakel KM, Larios O, Allen V, Lee B, Nadon C 2010 High-throughput genome sequencing of two Listeria monocytogenes clinical isolates during a large foodborne outbreak. BMC Genomics 11:120–135. doi:10.1186/1471-2164-11-120. 20167121 PMC2834635

[3] Gasanov U, Hughes D, Hansbro PM 2005 Methods for the isolation and identification of Listeria spp. and Listeria monocytogenes: a review. FEMS Microbiol Rev 5:851–875. doi:10.1016/j.femsre.2004.12.002. 16219509

[4] Cuccuru G, Orsini M, Pinna A, Sbardellati A, Soranzo N, Travaglione A, Uva P, Zanetti G, Fotia G 2014 Orione, a Web-based framework for NGS analysis in microbiology. Bioinformatics 30:1928–1929. doi:10.1093/bioinformatics/btu135. 24618473 PMC4071203

[5] Bankevich A, Nurk S, Antipov D, Gurevich A, Dvorkin M, Kulikov AS, Lesin V, Nikolenko S, Pham S, Prjibelski A, Pyshkin A, Sirotkin A, Vyahhi N, Tesler G, Alekseyev MA, Pevzner PA 2012 SPAdes: a new genome assembly algorithm and its applications to single-cell sequencing. J Comput Biol 19:455–477. doi:10.1089/cmb.2012.0021. 22506599 PMC3342519

[6] Assefa S, Keane TM, Otto TD, Newbold C, Berriman M 2009 ABACAS: algorithm-based automatic contiguation of assembled sequences. Bioinformatics 25:1968–1969. doi:10.1093/bioinformatics/btp347. 19497936 PMC2712343

[7] Nadalin F, Vezzi F, Policriti A 2012 GapFiller: a de novo assembly approach to fill the gap within paired reads. BMC Bioinformatics 13:S8. doi:10.1186/1471-2105-13-S14-S8. PMC3439727 23095524

[8] Piro VC, Faoro H, Weiss VA, Steffens MB, Pedrosa FO, Souza EM, Raittz RT 2014 FGAP: an automated gap closing tool. BMC Res Notes 7:371. doi:10.1186/1756-0500-7-371. 24938749 PMC4091766

[9] Walker BJ, Abeel T, Shea T, Priest M, Abouelliel A, Sakthikumar S, Cuomo CA, Zeng Q, Wortman J, Young SK, Earl AM 2014 Pilon: an integrated tool for comprehensive microbial variant detection and genome assembly improvement. PLoS One 9:e112963. doi:10.1371/journal.pone.0112963. 25409509 PMC4237348

[10] Langmead B, Salzberg SL 2012 Fast gapped-read alignment with Bowtie 2. Nat Methods 9:357–359. doi:10.1038/nmeth.1923. 22388286 PMC3322381

[11] Yoon SH, Ha SM, Lim J, Kwon S, Chun J 2017 A large-scale evaluation of algorithms to calculate average nucleotide identity. Antonie Van Leeuwenhoek 110:1281–1286. doi:10.1007/s10482-017-0844-4. 28204908

